# Chromatographic single-step purification of tagless proteins using gp41-1 split inteins

**DOI:** 10.3389/fbioe.2023.1319916

**Published:** 2024-01-17

**Authors:** Michael Knapp, Vanessa Kohl, Tatjana Best, Oliver Rammo, Sybille Ebert

**Affiliations:** ^1^ Faculty of Natural Sciences, Ulm University, Ulm, Germany; ^2^ Institute of Applied Biotechnology, Biberach University of Applied Sciences, Biberach, Germany; ^3^ Merck—Life Science KGaA, Darmstadt, Germany

**Keywords:** gp41-1, split intein, tagless purification, affinity chromatography, cleaning

## Abstract

The current trend in biopharmaceutical drug manufacturing is towards increasing potency and complexity of products such as peptide scaffolds, oligonucleotides and many more. Therefore, a universal affinity purification step is important in order to meet the requirements for cost and time efficient drug production. By using a self-splicing intein affinity tag, a purification template is generated that allows for a universal chromatographic affinity capture step to generate a tagless target protein without the use of proteases for further tag removal. This study describes the successful implementation of gp41-1-based split inteins in a chromatographic purification process for, e.g., *E. coli*-derived targets. The tagless target is generated in a single-step purification run. The on-column cleavage is induced by triggering a simple pH change in the buffer conditions without the need for additives such as Zn^2+^ or thiols. This system has proven to be reusable for at least ten purification cycles that use 150 mM H_3_PO_4_ as the cleaning agent.

## 1 Introduction

According to a recent survey, antibodies still account for the majority of biopharmaceutical drug approvals. Nevertheless, other modalities such as new nucleic acid-based therapeutics or cellular therapies are slowly making their way onto the market as well (Walsh, 2018). Due to its unique selectivity, the implementation of an initial affinity capture step using Protein A in a classical mAb production process was a key achievement. A cost- and time-efficient mAb production template was established (Bolton and Mehta, 2016) after more than 40 years of advancement since the introduction of the first commercial Protein A resin in 1978.

With regard to future biopharmaceutical market trends, novel modalities are expected to be a key growth pillar. These could include peptide scaffolds, oligonucleotides, hybrids, molecular conjugates and other molecules that tackle the resolution of current downstream solutions due to their high diversity and complexity (Valeur et al., 2017; Walsh, 2018). Therefore, a universally applicable downstream solution analogous to conventional Protein A would be desirable.

A tag-based affinity chromatography method that leads to purification yields higher than 90% could be one possibility. An affinity tag is typically cloned and expressed in frame together with the protein of interest (POI) either N- or C-terminally. The purification principle is based on the known interaction of the affinity tag and an immobilized ligand on the stationary phase, providing a simple platform for the purification of a broad range of molecules (Young et al., 2012; Lahiry et al., 2018).

In turn, the tag-based affinity chromatography also implies a general disadvantage of the tag itself. In some cases, the tag can affect the properties of the protein or, for example, trigger immunogenic reactions when injected in humans. In this case, the tag often needs to be removed from the target protein after the initial affinity chromatography step. Tag removal can be accomplished by proteases that recognize a specific sequence motif inserted between the POI and the tag. The most frequently used proteases for such an approach are enterokinase, factor Xa, thrombin, tobacco, etch virus (TEV) and human rhinovirus 3C protease (Zhao et al., 2013). Although these proteases are very specific, offsite target cleavage still remains a major issue. It leads to traces of individual amino acids that are still present on the processed POI that can either alter the pharmacokinetic profile of the drug or cause undesirable immunogenic reactions. The optimal working conditions for the proteolytic tag removal often need to be adjusted individually for each target molecule, implying that the cleavage process efficiency can be impaired or reduced. The utilization of proteases is also cost-intensive and often ends in a time-consuming optimization approach to ensure proper tag removal that meets certain economical standards. Not just for this reason, but also due to the fact that the tag and the proteases need to be removed from the POI in an additional purification step, the use of affinity chromatography in large scale biopharmaceutical manufacturing is faced with a limitation (Young et al., 2012).

A self-cleaving functionality of an affinity tag would allow for an affinity capture step without the need for costly and time-consuming protease treatment of the POI for proper affinity tag removal. The intein purification (internal protein) technology could provide a convenient solution to simplify current protein purification processes, especially for the initial capture step. The detailed protein splicing process of the inteins are already described by Lahiry et al. and Mills, Johnson and Perler (Mills et al., 2014; Lahiry et al., 2018).

Inteins can be used for various biotechnological applications such as biosensors, protein labeling, cyclization and purification (Sarmiento and Camarero, 2019). The development of self-splicing affinity tags to produce tagless proteins (Lahiry et al., 2018) became the first major field of application for inteins. Guan et al. developed an engineered *Npu* DnaE split intein system for tagless protein purification called SIRP (Split Intein Mediated Ultra-Rapid Purification of Tagless Protein) under consideration of the N-terminal and C-terminal splitted intein class (split inteins). Through various modifications of *Npu* DnaE, a chitin binding domain intein-N and an intein-C-POI were developed, whereby the chitin binding domain intein-N binds to a chitin-modified resin capable of capturing the intein-C POI. Through molecular engineering of the C1 position (C1A mutation), the splicing process could be abolished and replaced by a mechanism that only took place at the C-terminus of the intein called the C-terminal splicing mechanism (cleavage). Thus, tagless purification of the POI from a clarified *E. coli* cell lysate with >90% cleavage efficiency could be performed by applying reducing agents such as 50 mM dithiothreitol (DTT). Without the addition of the reducing agent DTT, only 50% cleavage was observed after 30 min at 22°C (Guan et al., 2013). One of the commercially available solutions is based on contiguous inteins such as the IMPACT™ system that requires about 16 h at 23°C to achieve comparable results. Although *Npu* DnaE has been found to be the most extein-tolerant protein with fast reaction kinetics, the addition of Zn2+ needed for the inhibition and DTT for the thiol induction of the cleavage reaction represents a major limitation, especially for the purification of proteins containing disulfide bonds (Guan and Chen, 2017). Other mechanisms that can trigger the N-, C-terminal or both-end splicing reaction besides the utilization of chemical inductors are light, temperature, salt concentration or pH (Lahiry et al., 2018), for example,.

In the Global Ocean Sampling (GOS) project, new split inteins such as gp41-1, gp41-8, NrdJ-1, and IMPDH-1 were discovered and engineered through the analysis of metagenomic data. With the cyanophage-like gp41-1 which descends from the gp41 DNA helicase, an ultra-fast intein for protein trans-splicing was discovered that exhibited around ten times higher reaction kinetics compared to the native *Npu* DnaE at 37°C and even 13 times higher at 45°C. The engineered gp41-1 can tolerate various buffer conditions without causing any unwanted side reactions, leading to C-terminal cleavage yields of typically 90%–95%. The ideal conditions for performing the intein-specific cleavage reaction were achieved at a pH of 7 and can be inhibited by increasing only the pH value. This implies that the addition of Zn2+ or DTT is no longer needed to trigger the intein-specific cleavage process. Thus, the gp41-1 has proven to be the most promising split intein with the highest reaction rate and efficiencies (Dassa et al., 2009; Carvajal-Vallejos et al., 2012). The recently developed iCapTagTM technology is a new commercially available solution from Protein Capture Science. The pH-inducible cleavage used is also based on modified segments of the *Npu* DnaE intein (Gierach and Wood, 2021). The usage of the modified *Npu* DnaE within the iCapTagTM system typically releases 90% target, whereas the target can be *E. coli*, CHO or HEK cell derived. This pH-sensitive enzymatic cleavage takes place at room temperature by reducing the pH from 8.5 to 6.2, whereas the cleavage time is comparatively low. Nevertheless, modified *Npu* DnaE segments as well as the fast gp41-1 intein generate a new level of biomolecule purification using intein based affinity chromatography without adding cleavage induction or inhibition agents (Gierach and Wood, 2021).

**FIGURE 1:**
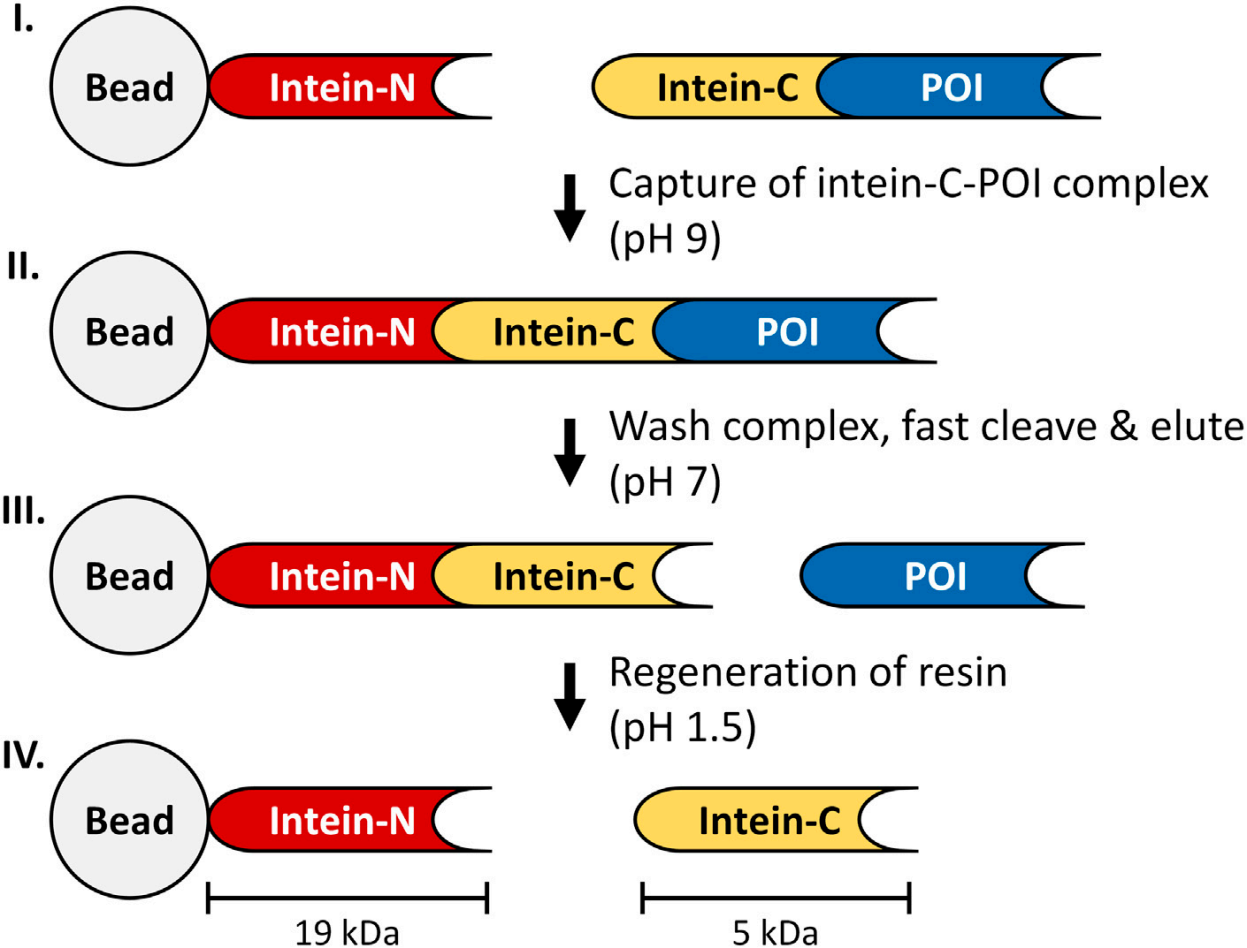
Principle of the intein-affinity chromatography. I: intein-N fragment is covalently bound to a bead, while the intein-C tag is expressed in frame with the POI. II: The immobilized intein-N fragment captures the intein-C POI to form a complex (pH 9), while impurities are washed away. III: The cleavage process is triggered by adjusting the buffer conditions to pH 7, releasing the tagless POI from the column. IV: intein-C tag is released from the column during regeneration of the resin (150 mM H_3_PO_4_, pH 1.5).

In this study, we describe the successful implementation of chromatographic single-step purification resulting in a tagless POI without the need for proteolytic processing based on an engineered gp41-1 split intein affinity column (Figure 1). The gp41-1 intein-N fragment was immobilized as affinity ligand on a chromatographic support that was packed and characterized in a 1 mL column format. The POI was expressed in frame with a C-terminal attached intein-C tag in the microbial expression system *E. coli*. The affinity of the intein-C tag and the intein-N fragment allowed the intein-C POI to be captured from the clarified cell lysate. The C-terminal cleavage of the POI was induced by simply changing the pH to neutral conditions in the elution buffer without requiring an inhibition agent such as Zn2+ or an inducing agent such as DTT as required in other systems (Guan et al., 2013). The implementation of this system as the initial capture step eliminates the need for cost-intensive protease treatment and additional purification steps for the removal of the affinity tag, as in conventional affinity chromatography that uses a His-tag or a Strep-tag^®^ II (Young et al., 2012). Furthermore, the successful cleaning of the column with 150 mM H_3_PO_4_ (pH 1.5) was demonstrated over the course of at least ten consecutive purification cycles with clarified *E. coli* cell lysate. H_3_PO_4_ as cleaning and sanitization solution replaces in fact the commonly used sodium hydroxide (NaOH) due to reducing the use of harsh solutions and a prevention of loss in ligand functionality. NaOH is by far the most used chemical for both cleaning and sanitization, whereas alternative chemicals may be used in manufacturing processes (Grönberg and Hjorth, 2018; Rammo and Skudas, (2021)).

## 2 Materials and methods

### 2.1 Equipment and chemicals

The chromatographic experiments were performed on a NGC™ Chromatography System (Bio-Rad Laboratories, Inc., Hercules, CA, United States) or an ÄKTA™ pure (Cytiva, Uppsala, Sweden) system. Each system was equipped with an UV monitor for measuring protein absorbance at 280 nm (A_280_). The column pressure was measured as the pre column pressure of the system (PreCol) and the additional pressure difference over the column bed (Δ-column pressure). Unless stated otherwise, all reagents and chemicals were purchased from Merck KGaA (Darmstadt, Germany).

### 2.2 Analytics

Aliquots of protein-containing solutions were optionally analyzed according to the purity level via SDS-PAGE and size exclusion chromatography (SEC). For the reducing SDS-PAGE, precast NuPAGE™ 4%–12% Bis-Tris gels (Thermo Fisher Scientific, Waltham, MA, United States) were used together with a 6.5–200 kDa SigmaMarker™ Wide Range or alternatively a 10–225 kDa Perfect Protein Marker (Merck KGaA, Darmstadt, Germany). Samples were denatured, loaded onto the gel and proteins were separated at 200 V for 25 min. The SEC analysis was performed using a BEH 200Å Guard column (4.6 mm × 30 mm, 1.7 µm) from Waters Corporation (Milford, MA, United States) in combination with a ACQUITY UPLC Protein BEH 200Å (4.6 mm × 150 mm, 1.7 µm) separation column from Waters. The protein samples were separated for 11 min at a contant flow rate of 0.3 mL/min using a 100 mM sodium phosphate and 100 mM sodium sulfate buffer at pH 6.7. The verification of the intact protein mass within the protein containing solutions was done via mass spectrometry (MS). Samples were analyzed by coupling a microflow LC system (M5, Eksigent) to an SCIEX ESI QTOF Mass Spectrometer system X500B (Framingham, MA, United States). Proteins were trapped for 1.5 min using the Luna reversed phase C18 (0.3 mm × 20 mm, 5 µm) trap column from Phenomenex (Torrance, CA, United States) at a constant flow rate of 50 μL/min with 95% solvent A (0.1% formic acid in water) and 5% solvent B (0.1% formic acid in Acetonitrile). Subsequently they were separated on a reversed phase C4 column (0.3 mm × 50 mm, 2.6 µm) from Waters using a 10 min gradient of 10%–70% solvent B at a constant flow rate of 10 μL/min. The column temperature was controlled at 35°C.

MS data were collected over a mass range of 500–3,500 Da with a spray voltage of 5400 V and a source temperature of 350°C.

### 2.3 Expression of the POIs

The model protein Thioredoxin 1 (TRX, UniProtKB: P0AA27, 13 kDa) was seen as a representative target protein from the *E. coli* proteome. The target was N-terminally tagged with an intein-C fragment (5 kDa) and C-terminally tagged with a Strep-Tag^®^ II (1 kDa). The cloning of the POI (intein-C-TRX-Strep, 18 kDa) into the expression plasmid pD451-SR was performed by an external supplier (Atum, Newark, CA, United States).

Chemically competent *E. coli* BL21 (DE3) cells (Merck KGaA, Darmstadt, Germany) were transformed with the POI containing pD451-SR expression plasmid and plated on an LB-agar (10 g/L Tryptone, 10 g/L NaCl, 5 g/L Yeast Extract, 15 g/L Agar) plate containing 30 μg/mL kanamycin overnight at 37°C. One single colony of *E. coli* BL21 (DE3) cells from the transformation plate was picked to inoculate a volume of 20 mL LB medium with 1 mM kanamycin in a 100 mL Erlenmeyer flask and cultivated overnight at 30°C and 200 rpm. For the main culture, a volume of 500 mL 50/10 medium (10 g/L Tryptone, 50 g/L Yeast Extract, 0.492 g/L MgSO_4_ × 7 H_2_O) in a 2.5 L Thomson Ultra Yield™ Flask with AirOTop™ Seal was inoculated with an aliquot of 10 mL of the preculture and cultivated at 37°C and 350 rpm. At an optical density (OD_600_) of 3-4, the expression of the POI was induced with 0.48 mM IPTG. Furthermore, 50 µL Antifoam 204 were added and the cells were cultured for 20 h at 20°C and 350 rpm post induction. Cell harvest was performed by centrifugation of the cell suspension in 500 mL Corning^®^ tubes for 25 min at 4°C and ×3,428 g. The supernatant was discarded and the pellets were stored at −80°C for further processing.

### 2.4 Sample preparation

Mechanical cell lysis was performed using a One Shot Cell Disruptor (Constant Systems Limited, Daventry, United Kingdom). The centrifuged biomass was thawed, resuspended with a 10-fold (w/v) mechanical lysis buffer (100 mM Tris, 150 mM NaCl, 5 mM MgCl_2_, pH 8) and homogenized in the homogenizer with one shot at 1 kbar. The resulting lysate was incubated for 10 min with 25 U/mL Benzonase^®^ and centrifuged for 25 min at 4°C and 20,800 x g. The supernatant was filtered through a 0.2 μm filter and used as clarified *E. coli* cell lysate for following Strep-tag-affinity chromatography or split intein-based affinity chromatography.

To generate a Strep-purified reference sample, the clarified *E. coli* cell lysate was passed through a Strep-Tactin^®^ column (IBA Lifesciences, Göttingen, Germany) with a column volume (CV) of 132 mL, washed with equilibration buffer (100 mM Tris, 200 mM NaCl, pH 9) and eluted in 100 mM Tris, 200 mM NaCl, 2.5 mM d-Desthiobiotin, pH 9. The detailed Strep-tag affinity chromatography protocol follows the manufacturers instructions and is described in Table 1. The concentration of the proteins was determined by absorbance at 280 nm (A_280_) and under consideration of the molecules specific extinction coefficient. The purified protein stock was used as a Strep-purified reference sample for following split intein-based purification.

**TABLE 1:** Protocol for the individual chromatographic purification process. Step-tag affinity chromatography or intein-affinity chromatography was performed on the Äkta™ and the NGC™ system. The Strep-affinity chromatography was performed at 10 mL/min flow rate, the intein-affinity chromatography at 0.2 mL/mL or a combination of 1 mL/min and 0.1 mL/min (elution) instead.

Method	Step	Buffer conditions	CV
Strep-tag affinity chromatograph 1CV = 132 mL	Equilibration	100 mM Tris, 200 mM NaCl, pH 9	2
	Sample Application	sample dependent	sample dependent
	Column Wash	100 mM Tris, 200 mM NaCl, pH 9	4
	Elution	100 mM Tris, 200 mM NaCl, 2.5 mM d-Desthiobiotin, pH 9	3
	Column Wash	100 mM Tris, 200 mM NaCl, pH 9	3
	CIP	100 mM Tris, 150 mM NaCl, 1 mM HABA, 1 mM EDTA, pH 8	3
	Column Wash	100 mM Tris	4
	Equilibration	100 mM Tris, 200 mM NaCl, pH 9	2
Intein-affinity chromatography 1CV = 1 mL	Equilibration	100 mM Tris, 200 mM NaCl, pH 9	5
	Sample Application		5
	Column Wash	100 mM Tris, 200 mM NaCl, pH 9	5
	Elution Phase 1	100 mM Tris, 200 mM NaCl, pH 7	4–6
	Static Incubation	—	60–120 min
	Elution Phase 2	100 mM Tris, 200 mM NaCl, pH 7	4–6
	CIP	150 mM H_3_PO_4_, pH 1.5	5

### 2.5 Split intein-based purification

#### 2.5.1 Static binding capacity

The in 2.4 purified intein-N proteins were immobilized to Epoxy-BDM (Merck Life Science KGaA, Darmstadt, Germany), to generate several different intein-N affinity resin prototypes (R1-R4). The prototype materials were used for a static binding capacity (SBC) assay in a high throughput format. The goal of this investigation was solely to determine the capacity of the resin. After loading the intein-C tagged target, the resins were directly subjected to cleaning conditions. Triggering the enzymatic cleavage reaction in an additional elution step that typically takes place before cleaning was avoided and not part of the investigation. The SBC was determined from the regeneration fractions that were collected during cleaning in place (CIP) step of the column and the resins were exposed to different test conditions before starting the assay. A 10% (v/v) resin slurry was incubated for 0, 2 or 15 h in 150 mM H_3_PO_4_ under shaking conditions at room temperature (RT). The resin was washed and resuspended with capture buffer (100 mM Tris, 200 mM NaCl, pH 9) to a final 10% (v/v) resin slurry. A sample size of 100 µL resin slurry was transferred to a 96-well filter plate. The resin was equilibrated with capture buffer and 200 μL of a Strep-purified reference sample containing the POI with c_POI_ = 1 mg/mL (preparation of the Strep-purified reference sample is described in 2.4) were added to the equilibrated resin for 1 h under shaking conditions (900 rpm at 20°C). Any residuals of unbound intein-C target were removed by washing the resin with the capture buffer. The release of the bound target was triggered by two regeneration buffers (first: 10 mM Glycine, pH 1. Second: 150 mM H_3_PO_4_, pH 1.5). The breakthrough wash and regeneration fractions were collected in 96-well UV plates and the protein amount of the fractions and static binding capacity of intein-N affinity resin prototypes were calculated using the absorption at 280 nm (A_280_) and the extinction coefficient of the target.

#### 2.5.2 Intein column purification

To test the functionality of the single-step purification mechanism on a column process, a POI was purified from a Strep-purified reference sample (described in 2.4). The purification was performed by intein-N affinity resin prototypes packed into 18.9 mm bed height x 8.3 mm inner diameter scout columns corresponding to 1 mL column volume (Table 1). The Strep-purified reference sample was diluted in capture buffer (100 mM Tris, 200 mM NaCl, pH 9) to achieve a stock concentration of c_POI_ = 1 mg/mL. To test the single-step purification of POI directly from the clarified cell lysate, the *E. coli* cell lysate prepared as described in 2.4 was used with a concentration of c_POI_ = 2 mg/mL or a diluted lysate with c_POI_ = 0.2 mg/mL, instead.

The purification steps were performed according to the following scheme (Table 1): the column was equilibrated with 5 CV capture buffer. A volume of 5 mL Strep-purified reference sample (c_POI_ = 1 mg/mL) or clarified *E. coli* cell lysate (c_POI_ = 0.2 mg/mL) was loaded onto the column and the column was washed with 5 CV of capture buffer. The elution of the tagless POI was triggered in three sequential phases: first, the application of 4–6 CV cleavage buffer (100 mM Tris, 200 mM NaCl, pH 7). Second, a static incubation phase for 60 or 120 min (flowrate = 0 mL/min). Third, the further addition of 4–6 CV cleavage buffer. After this elution sequence, the column was regenerated during the CIP step (CIP = cleaning in place) with at least 5 CV CIP buffer (150 mM H_3_PO_4_, pH 1.5). All phases were performed at a flowrate of 0.2 mL/min. For an additional regeneration study of the column, including ten consecutive cycles of purification using clarified *E. coli* cell lysate (prepared as described in 2.4), the flow rate was adjusted to 1 mL/min except for the elution phase that was performed at 0.1 mL/min. The cycles were performed sequentially without intermediate exposure to storage solution.

The regenerated column was stored at 20% ethanol and 150 mM NaCl. The elution fractions were analyzed for protein quantity by peak integration using an internal UV detector of the chromatography systems and the absorbance of the fractions at 280 nm. The elution yield was calculated as the protein amount that could be collected and recovered during the elution step. The total bound protein amount (binding capacity) was calculated by quantifying the protein amount in the elution and CIP step. The purity of the elution fractions was analyzed using orthogonal methods such as SDS-PAGE analysis.

The elution fractions of two reference runs using a Strep-purified reference sample and clarified *E. coli* cell lysate were analyzed for purity and identity by SEC and LC-MS, respectively.

The concentration of remaining host cell proteins (HCP’s) and bacterial endotoxins was determined from several representative elution fractions of the regeneration study by HCP ELISA (#F410, Cygnus Technologies, Southport, NC, United States) and LAL gel-glot assay (#N284-25, Lonza Group, Basel, Switzerland) according to the manufacturer’s instructions.

## 3 Results and discussion

### 3.1 Static binding capacity

**FIGURE 2:**
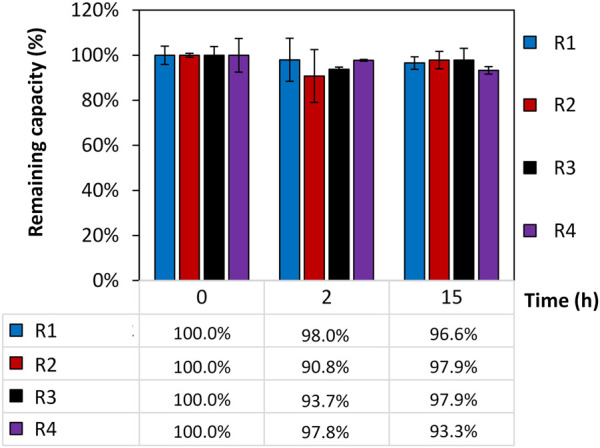
Remaining static binding capacity (SBC) of four intein-N affinity resin prototypes. The remaining SBC was calculated in percentages using the SBC at time point t = 0 SBC_t=0_ (R1) = 1.4 mg/mL; SBC_t=0_ (R2) = 1.6 mg/mL; SBC_t=0_ (R3) = 1.7 mg/mL; SBC_t=0_ (R4) = 3.1 mg/mL and the SBC after 0, 2, and 15 h exposure of the resins to 150 mM H_3_PO_4_ using the formula of SBC_t=x_/SBC_t=0_.

The remaining static binding capacity of four intein-N affinity resin prototypes (R1-R4) that were exposed to 150 mM H_3_PO_4_ for different time periods (0, 2, and 15 h) was calculated in triplicates, normalized, and presented in Figure 2. The capacity of the resins was recorded at time (t = 0) to achieve each resin’s initial SBC (t = 0). The SBCt = 0 for R1, R2, R3 and R4 was calculated to be 1.4 mg/mL, 1.6 mg/mL, 1.7 mg/mL and 3.1 mg/mL. Any changes of the initial SBC were recorded after exposure to H_3_PO_4_ for three time points 0/2/15 h. The changes are shown in percentages using the formula SBC_t=x_/SBC_t=0_. No significant loss of the resin’s SBC was observed when exposing the resin to 150 mM H_3_PO_4_ for up to 15 h and a capacity of 96.4% ± 3.6% could be calculated. A portion of uncoupled resin beads served as a control.

### 3.2 Intein column purification

**FIGURE 3:**
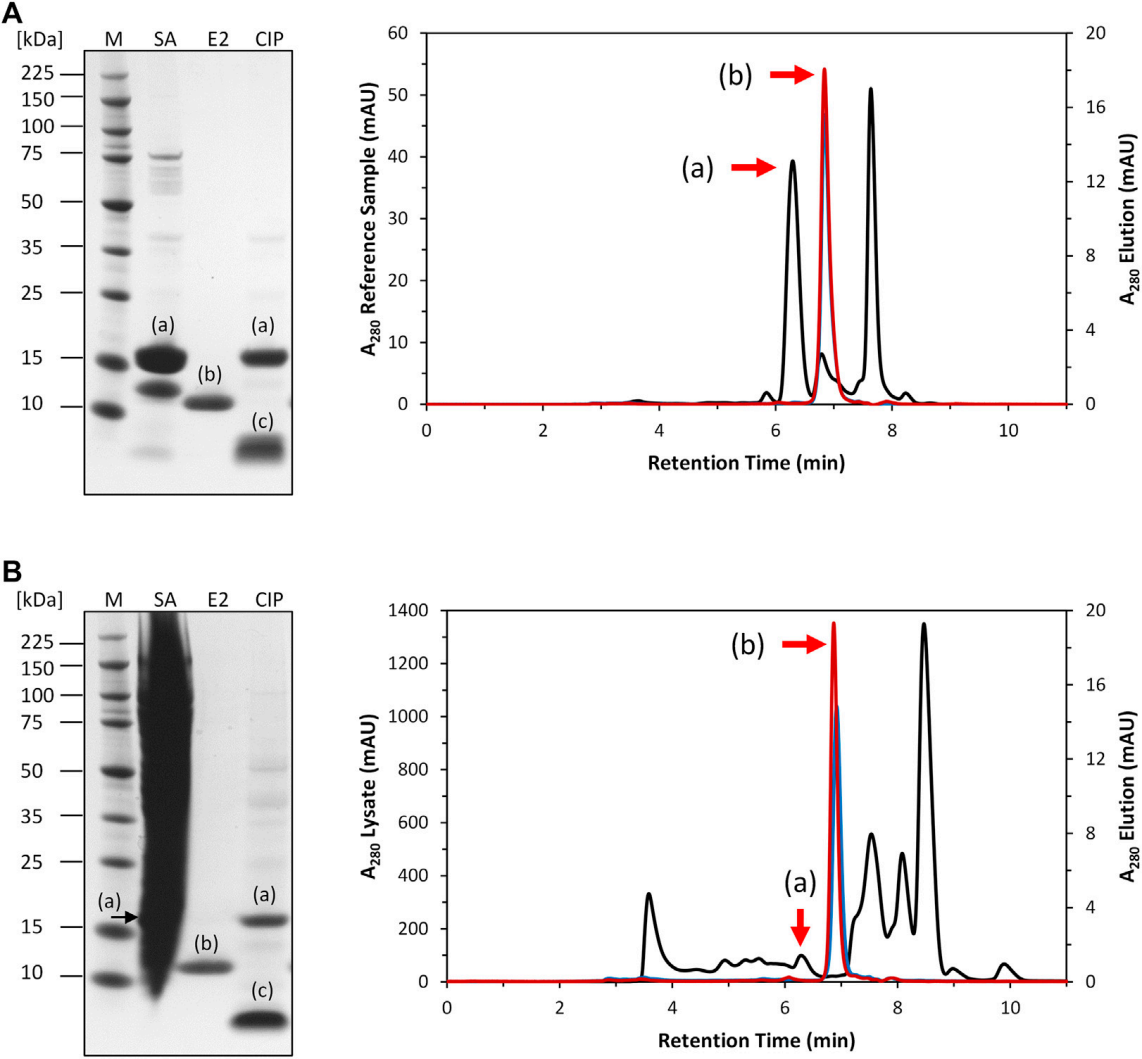
SDS-PAGE and SEC analysis of intein column purification. **(A)**: Purification of 5 mL Strep-purified reference sample (black). The tagless POI was recovered with a purity of 95.8% from the elution phase 1 (blue) and 97.4% from elution phase 2 (red). **(B)**: Purification of 5 mL clarified *E. coli* cell lysate (black). The tagless POI was recovered with a purity of 91.9% from elution phase 1 (blue) and 93.5% from elution phase 2 (red). (a) = intein-C POI. (b) = tagless POI. (c) = cleaved intein-C tag. SA = sample application. E2 = elution phase 2 fraction exemplary for the whole elution. CIP = regeneration.

After expression, the clarified *E. coli* cell lysate was purified by Strep-tag affinity chromatography to generate the Strep-purified reference sample (described in 2.4). A column containing the intein-N affinity resin prototype was loaded with a volume of 5 mL Strep-purified reference (c_POI_ = 1 mg/mL) sample Figure 3A). The elution fractions contained only the tagless POI at 13 kDa with high purity (>95.8%). The cleaved off intein-C tag could be recovered in the CIP fractions at 5 kDa together with any residual amount of uncleaved POI at 18 kDa. The total amount of eluted tagless POI was 0.5 mg, corresponding to an elution yield of 59.9%.

**FIGURE 4:**
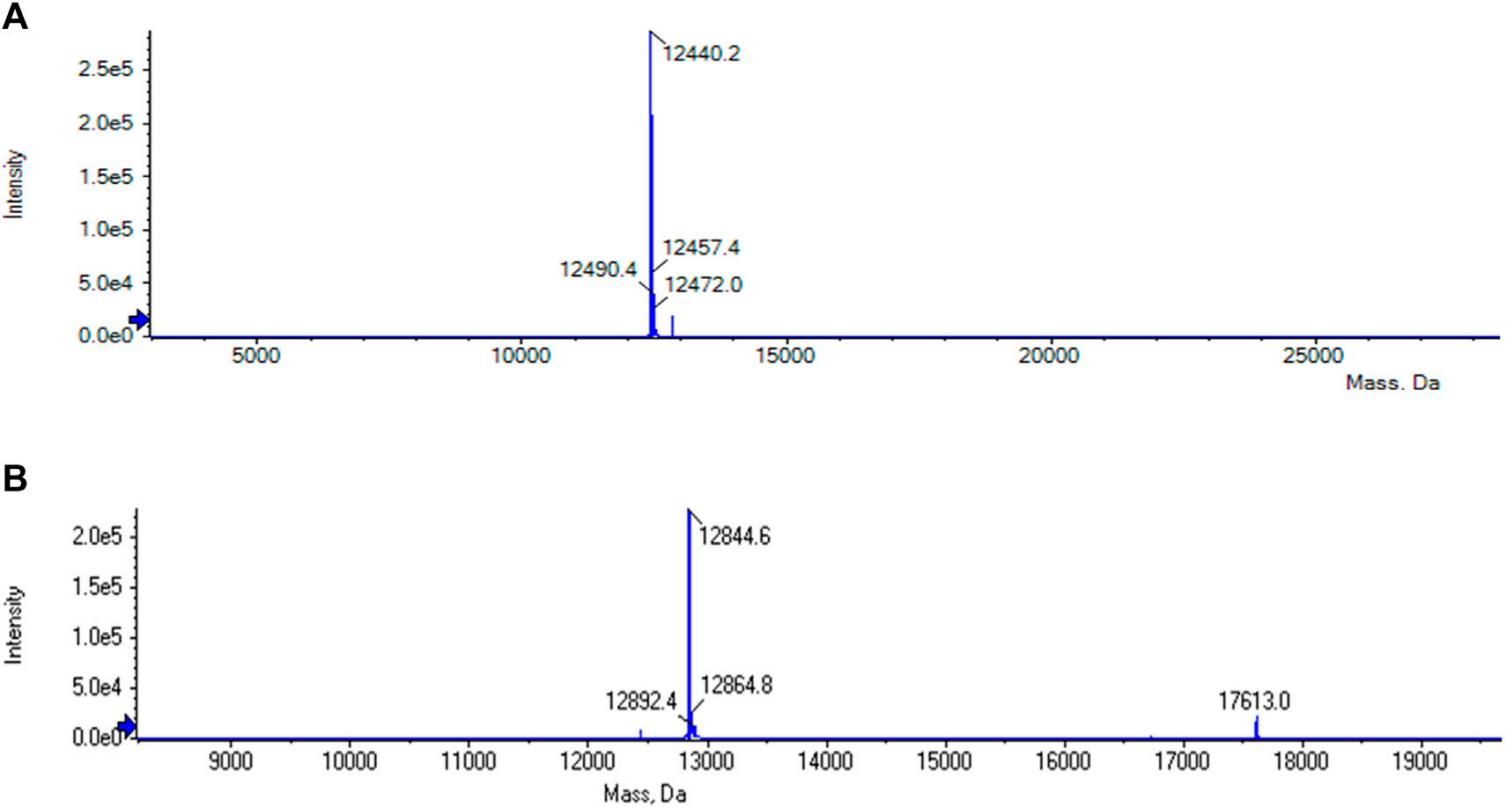
**(A)** MS analysis of the elution phase 2 fraction from the reference run using clarified *E. coli* cell lysate. The tagless POI with the loss of three C-terminal amino acids from the Strep-Tag^®^ II (amino acids F, E and K) could be found at 12.4 kDa. **(B)** MS analysis of the elution phase 1 fraction from cycle 1 of the regeneration study using clarified *E. coli* cell lysate. The tagless POI was visible at a molecular weight of approximately 13 kDa together with uncleaved POI at approximately 18 kDa.

The purification of the tagless POI was also investigated using a load volume of 5 mL of clarified *E. coli* cell lysate (c_POI_ = 2 mg/mL, Figure 3B). The elution fractions featured the tagless POI with the proper size at 13 kDa with a high purity level (>91.9%). The total amount of eluted tagless POI was 2.29 mg, corresponding to an elution yield of 70.6%. The exemplary electron ionization mass spectrum of the elution fraction, using clarified cell lysate for the purification, demonstrated the proper identity of the tagless POI with the loss of three C-terminal amino acids from the Strep-Tag^®^ II (amino acids F, E and K) at 12.4 kDa (Figure 4). Although the Strep-Tag^®^ II is typically resistant to cellular proteases, an obvious protein degradation of the tag over the whole process (including purification, storage and analysis) caused the loss of these three amino acids.

The fact that the target appears to be tagless at a purity level >90% in the elution fractions successfully demonstrates the functionality of the on-column capture and cleavage of the split intein-based purification system without the need for proteases or the induction or inhibition by DTT or Zn2+ (Guan and Chen, 2017). The column could be successfully cleaned by applying 5 CV 150 mM H_3_PO_4_ at pH 1.5 that regenerated the column. It included a composition of a residual amount of uncleaved POI and a majority of column-associated intein-C tag. Since the CIP fractions still contain a small portion of uncleaved target, one can assume that the reaction conditions are not yet finalized and that the elution yield could be further increased through further process adjustments.

### 3.3 Column lifetime study

For investigating the lifetime of a selected intein-N column carrying an intein-N affinity resin prototype, the performance of the column was studied for ten consecutive purification cycles. The column performance was investigated post-cleaning using a sample-volume of 5 mL diluted clarified *E. coli* cell lysate with a defined POI concentration (c_POI_ = 0.2 mg/mL) for each purification cycle. The column performance was investigated in relation to the amount of total bound protein together with the amount of eluted protein (elution yield).

**FIGURE 5:**
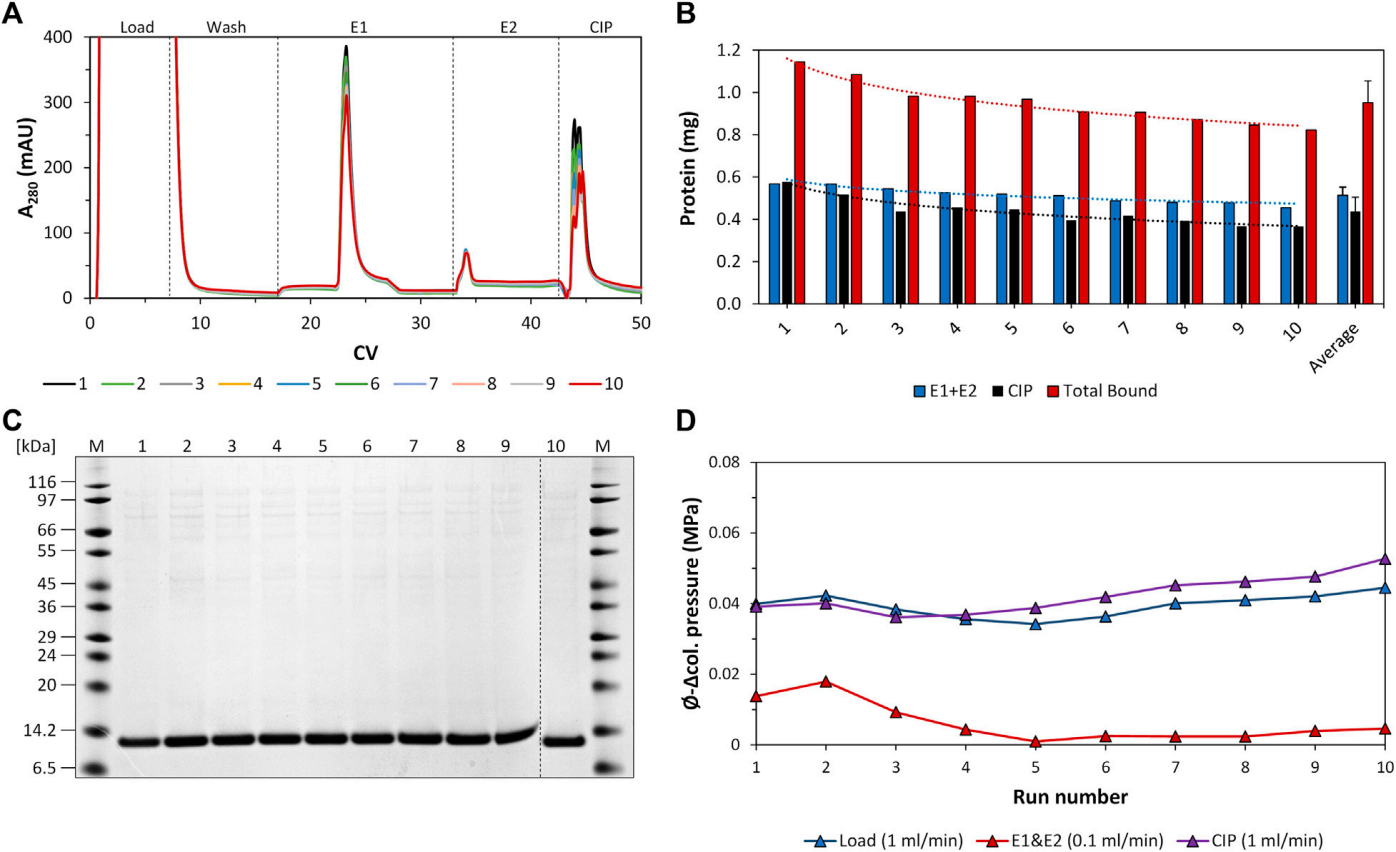
Regeneration study of the intein-N column using crude *E. coli* cell lysate. **(A)** Overlay of the respective UV profile at 280 nm of all ten consecutive purification cycles. The section was adapted to match the elution and regeneration phases (CIP phases). Peaks: Load, elution phase 1, elution phase 2, CIP phase. The flow rate of the load and CIP phases was 1 mL/min. The flow rate of the elution phases was 0.1 mL/min. **(B)** Protein amount recovered from the elution phases 1 and 2 (elution yield, blue), the CIP phase (black) and the total amount of bound protein (capacity, red) during the consecutive purification cycles and on average. The values for eluted protein amount and total bound protein are added additionally as numbers above the bars. **(C)** SDS-PAGE analysis of the elution phase 1 fractions of all consecutive purification cycles. **(D)** Average Δ-column pressure of the load (blue), elution (red) and CIP phases (purple) of all consecutive purification cycles. The maximum Δ-column pressure limit of the column was 0.6 MPa. The PreCol pressure of the system, which is the pressure that was subjected to the column, was calculated to be between 0.062–0.069 MPa at cycle 1 and cycle 10.

**FIGURE 6:**
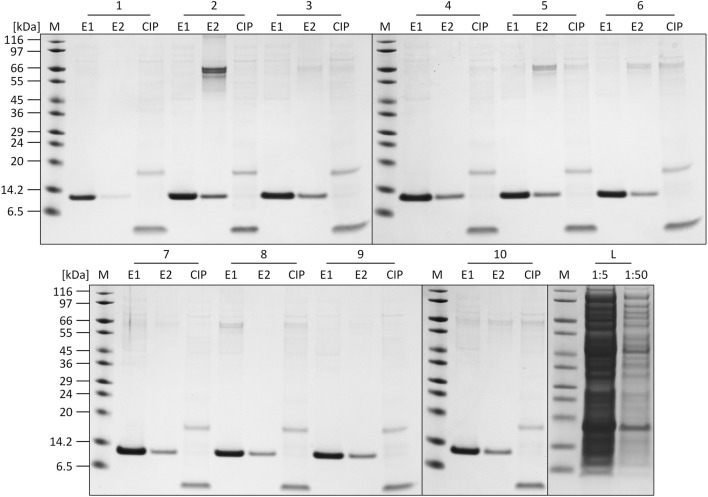
SDS-PAGE analysis of the elution (E1, E2) and CIP fractions of each cycle of the regeneration study. L = crude cell lysate loaded onto the column (1:5 and 1:50 dilution). M: SigmaMarker™ Wide Range.

The individual recorded UV profiles at 280 nm for the purification steps are displayed in Figure 5A, which shows the elution and CIP phases of each purification cycle in an overlay. Samples of the elution fractions of the ten consecutive purification runs were analyzed using SDS-PAGE analysis. The respective protein composition of the elution phase 1 fraction included one major protein band representing the proper size of the tagless POI (Figure 5C). According to SDS-PAGE analysis, these quality attributes do not change during ten consecutive purification runs. A detailed SDS-PAGE analysis comprising the recovered fractions of the elution phase 1, elution phase 2 and the CIP fractions, including the sample load, can be found in Figure 6. The PreCol and Δ-column pressure was recorded to monitor the cleaning efficiency of using 150 mM H_3_PO_4_. If blocking of the resin occurs, this would lead to an increase in the Δ-column pressure and an overall increase of the PreCol pressure over time and cycles, indicating resin damage or accumulated fouling.

For column loading, the PreCol pressure was measured to be constantly at 0.069 MPa for all cycles, for the CIP phase the PreCol reached from 0.062 to 0.069 MPa over all cycles. The Δ-column pressure over the course of all cycles ranged from 0.040 to 0.044 MPa for the loading phase and from 0.039 to 0.053 MPa for the CIP phase, respectively (maximum Δ-column pressure limit of the column at 0.6 MPa). This successfully demonstrates that regular cleaning of the column with 150 mM H_3_PO_4_ (Figure 5D) can prevent accumulation of fouling and extends the resin’s lifetime. The initial intein-N column binding capacity only dropped slightly from 1.15 mg initially to 0.82 mg within ten cycles of purification, resulting in an average column capacity of 0.95 ± 0.1 mg (Figure 5B). This effect was also observed for the elution yield, the recovered target only during the elution phases 1 and 2 of each purification cycle. The elution yield also shows a slight decline from 0.57 mg initially in cycle 1 to 0.45 mg in cycle 10, implying a decrease of 21.1%. The average protein amount over all ten consecutive purification cycles recovered from the elution phases 1 and 2 was 0.51 ± 0.1 mg.

The purity of the tagless POI within each elution phase 1 fraction was determined by performing intensity analysis of the protein band of the tagless POI in relation to all protein bands within the investigated lane (Figure 4). The average purity of the tagless POI in all ten consecutive purification cycles was 94% ± 2.5%. Some uncleaved POI could be found in the CIP fractions at 18 kDa. Therefore, the on-column reaction conditions are not yet optimal and the elution yield can potentially be increased further.

The purification of the lysate resulted in a 3.2-log reduction of the HCP and a 3.8-log reduction of the endotoxin concentration in the eluate. The eluate contained 0.1% ± 0.02% ppm HCP and the endotoxin level was calculated to be 2.5 × 105 EU/mg POI (Table 2).

**TABLE 2:** HCP and endotoxin concentration in the crude cell lysate and the elution fractions of the intein column purification process.

Sample	HCP concentration (ppm)	Standard deviation (ppm)	Endotoxin (EU/mL)	Log reduction HCP level	Log reduction endotoxin level
Lysate	169.56	0.45	1.0 × 10^8^	—	—
Elution	0.10	0.02	2.5 × 10^4^ (2.5 × 10^5^ EU/mgPOI)	3.2	3.8

## 4 Conclusion and outlook

The advantages of the intein technology discussed here include the simple implementation and ease-of-use to function as a single-step capture step for different targets out of clarified *E. coli* cell lysate, which prevents the need for time- and cost-intensive protease treatment. This study describes the successful implementation of an engineered gp41-1-based split intein in a chromatographic purification process for *E. coli*-derived targets. Since cleaning the column with NaOH resulted in a reduced loading capacity (data not shown), an acidic cleaning and sanitization solution was characterized. The intein column capacity can be restored by using 150 mM H_3_PO_4_ as the cleaning agent within a cleaning in place that results in remaining protein fragment release from the column and potential prevention of microbial and viral contaminations.

Considering the versatility of the intein-based purification technology and the increased complexity of molecules that are currently in the discovery phase, the intein technology could enable most cost-effective and productive process development and thus enable more molecules to reach the biopharmaceutical market. An investigation of microbial derived target purification could be demonstrated during this study. Additional experiments on highly glycosylated and mammalian derived model proteins, e.g., Erythropoetin (UniProtKB - P01588) could be successfully accomplished. The intein-based purification technology based on the gp41-1 intein could serve as a future universal purification template that allows for a simple and efficient single-step capture solution for a wide range of molecules from different expression systems. In latest studies, the technology was applicable for purification of mammalian derived intein-C tagged receptor binding domain (RBD) of the S1 spike glycoprotein of SARS-CoV-2. In contrast to conventional process schemes, multiple time-consuming and expensive chromatography steps downstream from the intein purification step can then be avoided. According to the multiple use of the technology for a broad range of molecules, the splicing reaction of gp41-1 shows another unique specification. It could be verified, that the cleavage can be induced by adding azole-like structures instead of pH triggered induction. A patent filed in 2021 focused on adapting processes to enable optimal and specific purification conditions, e.g., constant pH values (Rammo and Kohl, 2022), leading to a major advantage of this technology. By enabling pH-sensitive as well as additive-triggered enzymatic cleavage, a clear differentiation to other common systems can be highlighted. In the studies presented so far, the purification process and the total protein yield have been determined, whereas the investigation of the functionality or activity of the reference proteins before and after the purification step as well as a process optimization towards maximal binding capacity for the reference proteins was out of scope. Further investigations on novel intein-N affinity prototpye materials, could expand the highlights of this technology approach by generating higher binding capacities and increasing the overall productivity.

Besides process adaptability, the fast process itself could be demonstrated within that study. Whereas *Npu* DnaE based chromatographic materials show similar binding capacities and molecule recoveries, they differ in cleavage rate. The POI purification can be completed within several minutes in a dynamic mode with an optional additional incubation step accomplished in a second elution. This second elution step could be of interest for research purposes by enabling a fully enzymatic execution. In comparison, a minimum 5-h cleavage step at room temperature using the iCapTagTM method leads to longer process times. This results in a differentiation to this recently launched technology (Gierach and Wood, 2021).

Intein platform investigations used for chromatographic purification approaches could show cost and time savings as well as many more advantages, such as avoiding proteolytic tag removal and leftover non-native amino acids associated with the affinity tag that further need to be removed from the final product. Therefore, despite lower binding capacities than, for example, an ion exchanger, the intein technology could represent a convenient solution for significantly reducing purification costs for novel modalities and could well establish itself as a key invention for use in future biopharmaceutical drug manufacturing. However, as the technology is currently in development state, a direct and realistic cost comparison to existing purification approaches is nearly impossible. The greatest savings can be expected due to the lack of proteolytic tag removal and the use of simple buffers without expensive additives such as imidazole in the IMAC. In addition, the simple process should be mentioned here, which also results in time savings. Further optimization of the binding capacity and the maximum number of reuse cycles would make the technology even more cost-effective.

## Data Availability

The original contributions presented in the study are included in the article/Supplementary Material, further inquiries can be directed to the corresponding authors.
